# Epithelial arginase-1 is a key mediator of age-associated delayed healing in vaginal injury

**DOI:** 10.3389/fendo.2022.927224

**Published:** 2022-08-11

**Authors:** Holly N. Wilkinson, Benjamin Reubinoff, David Shveiky, Matthew J. Hardman, Ofra Ben Menachem-Zidon

**Affiliations:** ^1^ Centre for Biomedicine, Hull York Medical School, University of Hull, Hull, United Kingdom; ^2^ The Hadassah Human Embryonic Stem Cell Research Center and the Goldyne Savad Institute of Gene Therapy, Medical Center and Faculty of Medicine, Hebrew University of Jerusalem, Jerusalem, Israel; ^3^ Department of Obstetrics and Gynecology, Hadassah – Hebrew University Medical Center, Ein Kerem, Jerusalem, Israel; ^4^ Section of Female Pelvic Medicine & Reconstructive Surgery, Department of Obstetrics & Gynecology, Hadassah Medical Center, Faculty of Medicine, Hebrew University of Jerusalem, Jerusalem, Israel

**Keywords:** wound healing, arginase, vaginal repair, aging, prolapse (POP), estrogen

## Abstract

Pelvic organ prolapse is a disorder that substantially affects the quality of life of millions of women worldwide. The greatest risk factors for prolapse are increased parity and older age, with the largest group requiring surgical intervention being post-menopausal women over 65. Due to ineffective healing in the elderly, prolapse recurrence rates following surgery remain high. Therefore, there is an urgent need to elucidate the cellular and molecular drivers of poor healing in pelvic floor dysfunction to allow effective management and even prevention. Recent studies have uncovered the importance of Arginase 1 for modulating effective healing in the skin. We thus employed novel *in vitro* and *in vivo* vaginal injury models to determine the specific role of Arginase 1 in age-related vaginal repair. Here we show, for the first time, that aged rat vaginal wounds have reduced Arginase 1 expression and delayed healing. Moreover, direct inhibition of Arginase 1 in human vaginal epithelial cells also led to delayed scratch-wound closure. By contrast, activation of Arginase 1 significantly accelerated healing in aged vaginal wounds *in vivo*, to rates comparable to those in young animals. Collectively, these findings reveal a new and important role for Arginase 1 in mediating effective vaginal repair. Targeting age-related Arginase 1 deficiency is a potential viable therapeutic strategy to promote vaginal healing and reduce recurrence rate after surgical repair of pelvic organ prolapse.

## Introduction

Pelvic floor dysfunction encompasses a group of disorders that remain a significant clinical problem, reducing patient quality of life and affecting up to 25% of women in the United States alone (1, 2). One such disorder, pelvic organ prolapse (POP), occurs in as many as 50% of parous women (3). The most significant risk factor for POP is older age, where prevalence is highest in women aged between 60-69, with estimated lifetime risk of primary surgery reaching 20% by 80 years (2, 4). Surgical methods used to treat POP include lifting and supporting the pelvic organs (e.g. stitching or inserting a vaginal mesh graft), sometimes with a need for hysterectomy. Due to the link between POP severity and age (5, 6), and the largely elderly demographic for POP surgery (2), recurrent prolapse and re-operation is reportedly as high as 30% (3, 7). The number of women affected by POP continues to rise due to the expanding elderly population (8), contributing to increasing healthcare costs and patient morbidity (9). There is an urgent need to better understand the cellular and molecular perturbations that lead to prolapse, in order to effectively prevent and manage pelvic dysfunction.

It is established that the highest risk factors for POP are increased parity and older age. In addition, it is widely acknowledged that ageing leads to loss of tissue elasticity and degradation of extracellular matrix (ECM) proteins, resulting in morphological and biomechanical changes in tissues (10). Female biological aging is exacerbated by loss of sex hormone in women (menopause). Specifically, the rapid decline in levels of estrogen post-menopause may play a role in the pathogenesis of POP and in the suboptimal success of POP surgery (11). It is also clear that pelvic organs and their surrounding muscular and connective tissue support display broad estrogen receptor expression and are estrogen responsive (12). Age/estrogen-related tissue deterioration contributes to prolapse by weakening the levator ani muscle groups, uterosacral and cardinal ligaments and fibromuscular connective tissue of the vagina (13). Increased age and estrogen-deficiency is also a primary risk factor for poor tissue repair in rodents and humans (14–17). Thus, age-related defective healing may play a role in prolapse recurrence following surgery (18).

Aged cutaneous wounds show reduced re-epithelialisation, delayed ECM deposition and impaired inflammatory responses in both animal models and humans (14, 19, 20). Macrophages are key in governing wound inflammation, showing high plasticity and a diverse spectrum of phenotypes to enable effective tissue restoration (21). In general, classically activated “pro-inflammatory” macrophages are known to produce high levels of inducible nitric oxide synthase (iNOS), while alternatively activated “anti-inflammatory” macrophages, which are required for tissue resolution, produce elevated Arginase 1 (Arg1) (22).

Excessive production of *iNOS*, and reduced expression of *ARG1*, are associated with delayed healing in the elderly (23), while specific ablation of dermal Arg1 impairs skin repair (24). Much of the focus of Arg1 in wound healing is related to its macrophage-specific effects, yet recent findings have revealed a role for epidermal Arg1 in modulating effective skin repair (25). Given the number of studies addressing ageing and Arg1 in skin healing, it is surprising that reference to the contribution of these factors to repair in other tissues remains limited (13). Hence, the hypothesis for the present work is that epithelial Arg1 is essential for the timely repair of vaginal tissue and will be perturbed in delayed healing aged wounds. Here we explore, for the first time, the role of Arg1 in vaginal repair in young and aged rats, and in human vaginal epithelial cells. The data elucidate an important role for epithelial Arg1 in mediating effective vaginal repair, contributing to our understanding of prolapse recurrence following surgical healing failure.

## Materials and methods

### Animal experimentation

Young (12 weeks) and old (12 or 16 months) female Sprague-Dawley rats (n=63) were housed under constant temperature and humidity with a 12 hour light:dark cycle and *ad libitum* access to normal rodent diet and water. All experiments were approved by the Hebrew University Animal Care and Use Committee. Animals were anaesthetised with a mixture of Ketamine and Dormitor (75 mg/kg BW and 0.5 mg/kg BW, respectively). Upon completion of the procedure, administration of Atipamezole (1mg/kg) was given to reverse the anaesthetic effects. Vaginal wounds were generated by creating an 8 mm midline incision in the posterior vaginal wall, including the epithelial and posterior vaginal fibromuscular layers. For arginase activation experiments, vehicle (saline; 500 µL) or daidzein (7-Hydroxy-3-(4-hydroxyphenyl)chromone; MilliporeSigma, MO, US; 150 μg/kg) were administered intravenously to the tail vein to 16 month old animals. Animals were monitored daily until termination using overdose of CO_2_ and bilateral thoracotomy (as per the recommendations of the Panel on Euthanasia of the American Veterinary Medical Association). Tissue was collected for histological analysis and biomechanical testing as described below.

### Histological analysis

Vaginas from young and aged rats were fixed in 4% paraformaldehyde at days 1, 3 and 7 post-injury for comparative histology (n=3-7 per group). For arginase activation experiments, aged rat tissue was collected at day 3 post-injury (n=6 per group). Following embedding, sections (6 μm) were stained with haematoxylin and eosin and imaged on an Olympus BX61 microscope at 10X magnification. Wound closure measurements were performed by measuring the migration distance of the wound edge neo-epithelium as a percentage of total wound length. For immunofluorescent staining, sections were incubated in goat anti-Arg1 (N-20, Santa- Cruz Biotechnology, TX, US), rabbit anti-iNOS (Polyclonal, Novus Biologicals, CO, US) and rabbit anti-Ki67 (SP6, Cell Marque, MilliporeSigma) primary antibodies followed by appropriate Alexa Fluor conjugated secondary antibodies (all from Jackson ImmunoResearch Europe Ltd, Cambridgeshire, UK). Samples were counterstained and mounted with VECTASHIELD^®^ Antifade Mounting Media with 4′,6-diamidino-2-phenylindole (DAPI; Vector Laboratories, CA, US). Fluorescent images were captured on an Olympus BX61 microscope. Analysis was performed by counting positively stained cells per mm^2^ of tissue.

### Biomechanical testing

Full thickness vaginal tissue was carefully excised at 30 days post-injury for comparisons between young versus aged (n=5 per group), and vehicle versus daidzein (n=3 per group) groups. The vagina in its full thickness was carefully dissected from the surrounding structures and was longitudinally incised in its anterior wall along the urethra, forming a standardized rectangular specimen sized 1x2 cm. Each sample was clamped onto the mechanical analyser (Ta Instruments, New Castle, DE) and tension gradually increased until breaking point was reached. Data on force at breaking point (N) was then collected.

### Vaginal epithelial cell culture

Human vaginal epithelial cells (VK2/E6E7) were purchased from the ATCC and cultured in keratinocyte SFM (Gibco, Thermo Fisher Scientific, Leicester, UK) with 0.4 mM CaCl_2_ (MilliporeSigma), bovine pituitary extract, human recombinant epidermal growth factor and 1% (v/v) penicillin-streptomycin solution (all Gibco). For all experiments, VK2 cells were incubated at 37C with 5% CO_2_ and 95% humidity.

### Scratch wounding and treatments

Confluent monolayers of VK2 cells were scratched using a sterile 1 mL filter tip. Media was removed and cells rinsed in Dulbecco’s phosphate-buffered saline (Gibco) before adding fresh media. Endogenous Arg1 expression was first assessed in non-scratched (NS) versus scratched (S) cells. In follow-on experiments, Arg1 was blocked using 200 µM nor-NOHA (Biotechne, Oxford, UK). VK2 cells were treated with nor-NOHA for 6 hours (qRT-PCR) or 24 hours (immunofluorescence and arginase activity assay) depending on the assay.

### Quantitative real-time PCR

RNA was extracted from VK2 cells using TRIzol:chloroform phase separation as previously described (26). RNA was purified and eluted using the PureLink™ RNA Mini Kit (Thermo Fisher Scientific) following manufacturer’s instructions. GoScript™ and random primers (both Promega, Southampton, UK) were used for reverse transcription. cDNA was diluted in nuclease free water and primer sets amplified using 2x Takyon SYBR mastermix (Eurogentec, Hampshire, UK). qRT-PCR was performed on a CFX connect thermocycler (Biorad Laboratories Ltd., Hertfordshire, UK). Primer sequences are provided in **Supplementary Table S1**.

### Immunocytochemistry

VK2 cells were fixed in 4% (v/v) paraformaldehyde in Dulbecco’s phosphate-buffered saline for 15 minutes at room temperature. Cells were permeabilised with 0.01% Triton X-100, blocked with 1% bovine serum albumin and incubated in rabbit anti-Arginase 1 (PA5-29645; Invitrogen, Thermo Fisher Scientific) or mouse anti-Ki67 (MM1, Leica, Milton Keynes, UK) primary antibodies overnight at 4C. Detection was achieved using Alexa Fluor 488 conjugated secondary antibodies (Invitrogen). Rhodamine phalloidin and DAPI (both Invitrogen) were used to counterstain the cytoskeleton and cell nuclei, respectively. Imaging was performed using an LSM 710 confocal microscope (Carl Zeiss, Cambridge, UK) with 405 nm diode, 488 nm argon and 561 nm diode lasers. Intensity of staining (corrected total cell fluorescence) (27) was measured from 30 cells per image, across 30 images per group (three cell passages). Cell counts were performed across entire frames in ImageJ software v.1.8.0 (National Institutes of Health, Bethesda, MD).

### Arginase activity assay

Arginase activity was assessed using a modified version of (28). VK2 cells were lysed with 0.1% Triton X-100. Cell lysates and urea standards were added to an equal volume of arginase activation solution (10 mM MnCl_2_ and 50 mM Tris, pH 7.5) and incubated at 55C for 10 minutes. An equal volume of arginine substrate solution (0.5M L-arginine, pH 9.7) was then added and samples were incubated for 1 hour at 37C. To stop the reaction, 400 µL acid misc (1:3:7 H_2_SO_4_:H_3_PO_4_: dH_2_O) was added to 50 µL of sample, followed by 25 µL of 9% α-isonurosoprophenone. Samples were then incubated in the dark at 100C for 45 minutes, cooled, and absorbance read at 570 nm. Sample absorbance was compared to the urea standards and expressed as mg/ml.

### Livecyte ptychographic imaging

The Livecyte ptychographic imaging platform (Phase Focus, Sheffield, UK) was used to assess VK2 scratch wound closure over time in a label-free manner (29). Cells were imaged over a 24-hour period at 37C and 5% CO_2_. Percentage closure was deduced by measuring open scratch area in ImageJ. The Livecyte Cell Analysis Toolbox software segmented and tracked cells throughout the time-lapse, allowing additional automated measurement of average scratch area half-life and collective migration.

### Statistical analysis

Data are presented as mean +/- standard deviation of the mean (SEM). Independent *t* tests, Kruskall Wallis and Two-way ANOVA was performed on this data using R Studio. Tukey’s *post-hoc* analysis was used where appropriate. Significance was determined where *P* < 0.05.

## Results

### Delayed healing in vaginal wounds of aged rats is associated with significantly decreased Arginase 1 expression

**Figure 1:**
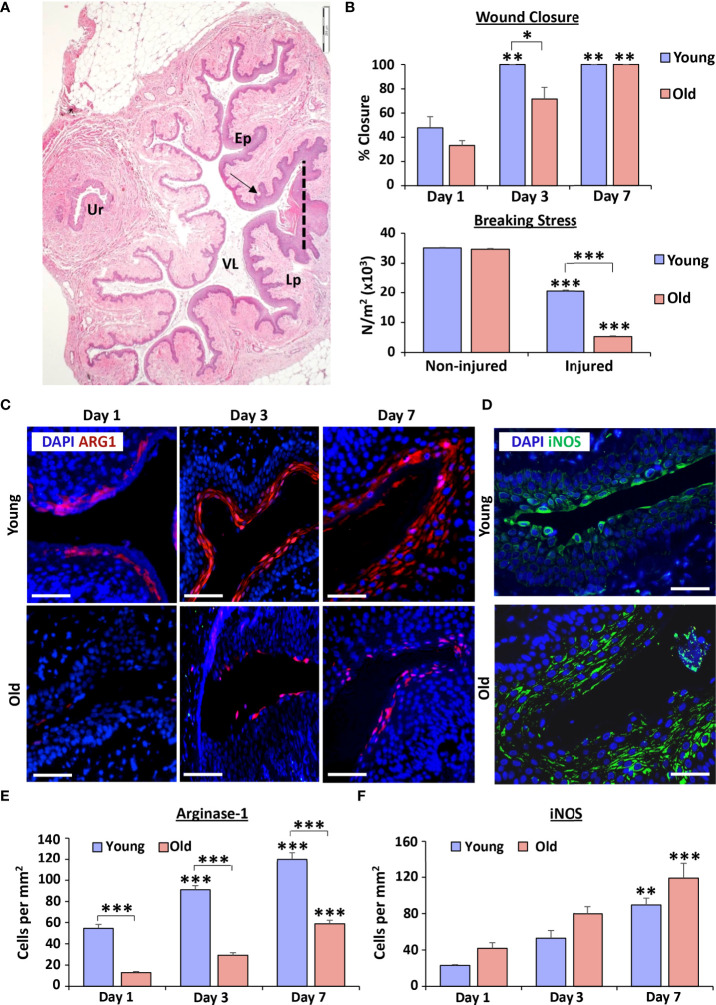
Ageing delays vaginal epithelial repair and significantly reduces Arginase 1 expression. Haematoxylin and eosin staining demonstrates rat vaginal tissue structure of a young rat at day 3 post-injury **(A)**. Ur = urethra, VL = vaginal lumen, Ep = epithelium, Lp = lamina propria. Dotted line = injury site. Vaginal epithelial wound closure rates between young (12 weeks) and old (12 months) rats over time **(B)**. Vaginal tissue breaking stress was assessed at 30 days post-injury **(B)**. n=5 rats per group. Arginase 1 (Arg1; **C**) at days 1, 2 and 7 post-wounding and iNOS **(D)** staining of vaginal epithelium at day 3 post-injury. Arg1 = Alexa Fluor 594. iNOS = Alexa Fluor 488. DAPI = nuclei. Bar = 200 µm. Quantification of Arg1 **(E)** and iNOS **(F)** staining. n = 3-7 rats per group. Mean +/- SEM. Kruskall Wallis and Two-way ANOVAs with Tukey *post-hoc* analyses were performed. * = P < 0.05, ** =*P* < 0.01 and *** =*P* < 0.001. Asterisk alone versus day 1 or non-injured within group.

A vaginal injury model was employed to evaluate the localisation and level of key Arginase/Nitric oxide pathway components in age-related delayed healing *in vivo* (**Figure 1**). In this model, vaginal wounds of aged rats showed significantly delayed closure compared to young rats at day 3 post-injury (*P* < 0.05; **Figure 1B**). Indeed, young rat wounds reached 100% closure (histological re-epithelialisation) by day 3 post-injury (*P* < 0.01) while aged rat vaginal wounds did not reach full closure until day 7 post-injury (*P* < 0.01). To assess the effects of ageing on the integrity of the reformed vaginal tissue, biomechanical testing was performed at day 30 post-injury. Here, breaking stress (N/m^2^) was 75% lower in the healed wounds of aged versus young rats (*P* < 0.001; **Figure 1B**). We next measured the levels of Arg1 in vaginal wounds due to its pertinent roles in skin repair (24, 25). Throughout the course of healing, epithelial Arg1 expression increased, with the highest levels observed in both young and aged wounds at day 7 post-injury (*P* < 0.001). However, epithelial Arg1 was significantly reduced in the aged model (versus young) at all healing time points (*P* < 0.001; **Figures 1C, E**). By contrast, although iNOS was upregulated in young (*P* < 0.01) and aged (*P* < 0.001) wound epithelium at day 7 post-injury (versus day 1), no difference in iNOS was observed between young and aged wounds at any time point (**Figures 1D, F**). Together, these data demonstrate that vaginal repair is significantly delayed in aged wounds, accompanied by substantial reduction in epithelial Arg1.

### Arginase 1 is induced upon injury in human vaginal epithelial cells

**Figure 2:**
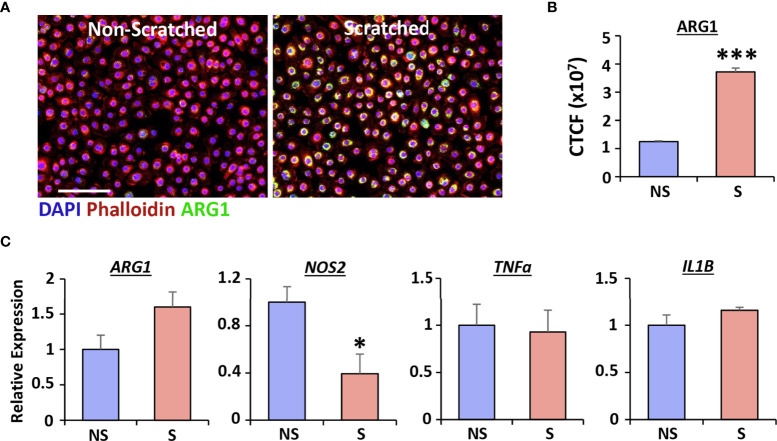
Injury induces Arginase 1 expression in human vaginal epithelial cells. qRT-PCR for *ARG1, NOS2*, *TNFα* and *IL1B* (**A**, n = 6). Immunofluorescence of Arginase 1 (ARG1) in non-scratched (NS) and scratched (S) cells **(B)** and quantification **(C)**. ARG1 = Alexa Fluor 488. Rhodamine phalloidin = cytoskeleton. DAPI = cell nuclei. Bar = 100 µm. CTCF = corrected total cell fluorescence. Mean +/- SEM. Independent *t* tests performed. *=*P* < 0.05 and ***=*P* < 0.001.

As Arg1 strongly correlates to healing following vaginal injury *in vivo*, we next used human vaginal epithelial cells to determine whether Arg1 was directly linked to injury *in vitro*. Wounds were created in human vaginal epithelial cells using the scratch wounding method and levels of Arg1 and inflammatory cytokines assessed in scratch-wounded versus non-scratched cells (**Figure 2**). Arg1 protein levels, evaluated *via* immunofluorescent staining, were significantly increased at 24 hours post-injury in human vaginal epithelial cells (*P* < 0.001; **Figures 2A, B**). The findings were confirmed by qRT-PCR which demonstrated a strong trend towards increased *ARG1* following injury (*P* = 0.07), accompanied by a significant decrease in *NOS2* (*P* < 0.001; **Figure 2C**). These changes in constituents of the Arg/iNOS pathway were not accompanied by changes in the inflammatory markers, *TNFa* or *IL1B*.

### Blockade of arginase 1 alters MMP expression and delays vaginal epithelial repair

**Figure 3:**
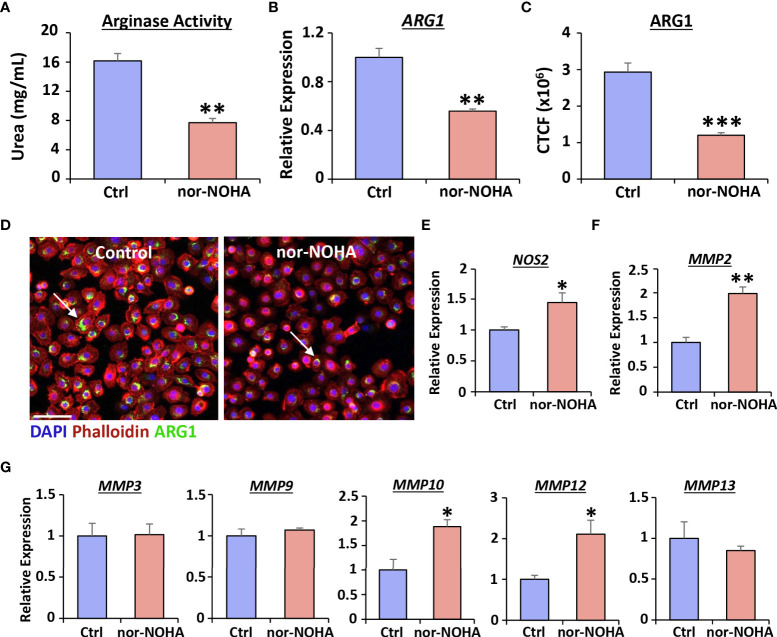
Nor-NOHA inhibits Arginase 1 and alters cytokine expression in human vaginal epithelial cells. An arginase activity assay was performed in VK2 cells following 24hr exposure to nor-NOHA **(A)**. Arginase 1 (ARG1) was assessed *via* qRT-PCR **(B)** and immunofluorescence **(C)**. CTCF = corrected total cell fluorescence. Images in **(D)** ARG1 = Alexa Fluor 488. Rhodamine phalloidin = cytoskeleton. DAPI = cell nuclei. Bar = 100 µm. qRT-PCR for *NOS2* **(E)**, *MMP2* **(F)** and other MMPs shown **(G)**. Mean +/- SEM. Independent *t* tests performed. *=*P* < 0.05, **=*P* < 0.01 and ***=*P* < 0.001.

To address the functional significance of Arg1 induction during vaginal epithelial repair, we used the specific Arg1 inhibitor, nor-NOHA, and assessed the subsequent healing effects in human cells. We first confirmed that treatment with nor-NOHA significantly inhibited Arg1 activity (*P* < 0.01; **Figure 3A**), *ARG1* expression *via* qRT-PCR (*P* < 0.01; **Figure 3B**) and ARG1 protein levels (*P* < 0.001; **Figures 3C, D**) in human vaginal epithelial cells. Arg1 inhibition also led to specific increase in expression of *NOS2* (*P* < 0.05; **Figure 3E**), *MMP2* (*P* < 0.01; **Figure 3F**), *MMP10* (*P* < 0.05) and *MMP12* (*P* < 0.05; **Figure 3G**).

**Figure 4:**
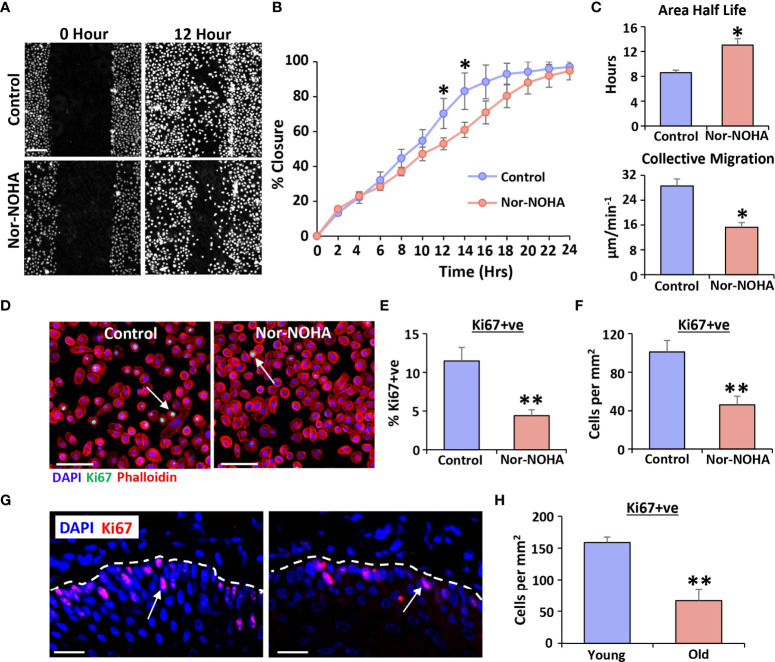
Arginase 1 inhibition delays healing in human vaginal epithelial cells and attenuates proliferation *in vivo*. Livecyte ptychographic imaging was performed to assess migration. Representative frames at 12 hours **(A)**. Bar = 100 µm. Quantification of closure over time **(B)**, average scratch area half-life and collective migration **(C)**. Ki67 staining for proliferation **(D)** and quantification **(E, F)**. Ki67 = Alexa Fluor 488. Rhodamine phalloidin = cytoskeleton. DAPI = cell nuclei. Bar = 100 µm. Ki67 staining in young and old rat vaginal epithelium at day 3 post-injury **(G)** and quantification **(H)**. Ki67 = Alexa Fluor 594. DAPI = cell nuclei. Bar = 100 µm. Mean +/- SEM. Two-way ANOVA with Tukey post-hoc in **(B)**. Independent *t* tests performed in **(C–F)**. * = P < 0.05 and ** = P < 0.01.

Label-free high-contrast live cell imaging (29) was next used to determine effect of blocking Arg1 on scratch wound closure in human vaginal epithelial cells. Overall wound closure was significantly delayed in nor-NOHA treated cells, reaching a peak difference to controls at 12-14 hours post-injury (*P* < 0.05; **Figures 4A, B**). This delayed migration was mirrored in other parameters of wound closure, with >50% increase in area half-life following nor-NOHA treatment (*P* < 0.05), accompanied by >50% decrease in collective migration velocity (*P* < 0.05; **Figure 4C**). In addition, nor-NOHA treatment significantly reduced vaginal epithelial cell proliferation (Ki67+ve cells; *P* < 0.01; **Figures 4D–F**). Turning to our *in vivo* vaginal injury model, we observed that aged rat wounds were not only deficient in Arg1 but showed substantially lower vaginal epithelial proliferation than young rat wounds at day 3 post-injury (*P* < 0.01; **Figures 4G, H**). Thus, our data strongly suggest a key role for Arg1 in regulating proliferative and migratory functions of epithelial cells following vaginal injury.

### Treatment with an arginase activator, Daidzein, is sufficient to restore vaginal repair in aged rats

**Figure 5:**
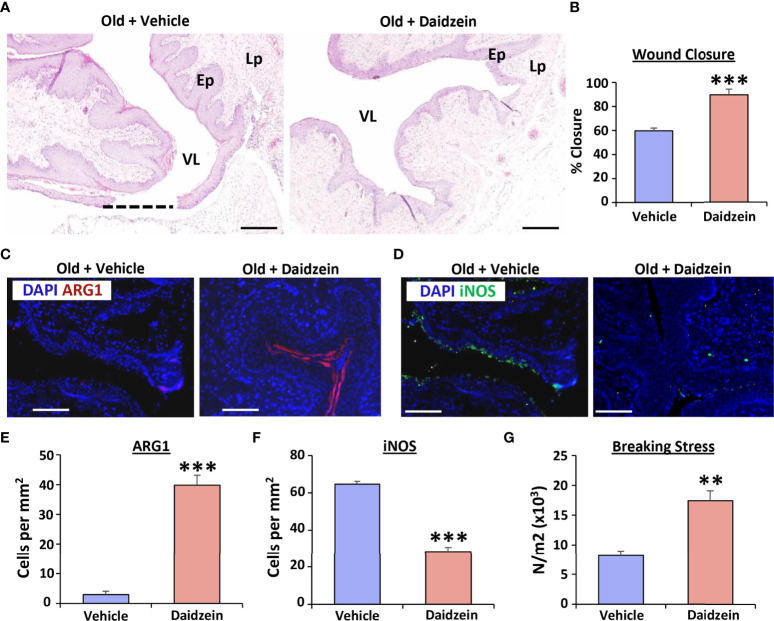
Daidzein activates Arginase I and restores vaginal repair in aged rats. Haematoxylin and eosin staining showing vaginal healing differences between vehicle and daidzein treated aged rats **(A)**, with quantification of wound closure in **(B)** VL = vaginal lumen, Ep = epithelium, Lp = lamina propria. Dotted line = open wound area. Bar = 500 μm. Arginase 1 (Arg1; **C**) and iNOS **(D)** staining of vaginal epithelium with quantification **(E, F)**. Arg1 = Alexa Fluor 594. iNOS = Alexa Fluor 488. DAPI = nuclei. Bar = 200 µm. n=6 rats per group. Vaginal tissue breaking stress, assessed 30 days following injury **(G)**. n = 3 rats per group. Mean +/- SEM. Independent *t* tests performed. **=*P* < 0.01 and ***=*P* < 0.001.

We next asked whether *in vivo* activation of arginase could reverse the delayed vaginal healing phenotype observed in aged rats (**Figure 5**). Here, daidzein, a small molecule transcriptional inducer of Arg1 (30), significantly accelerated vaginal wound closure in aged rats (*P* < 0.001; **Figures 5A, B**) by day 3 post-injury. Indeed, daidzein-treated wounds approached 100% closure, mimicking healing rates observed in young rats, while vehicle-treated aged rat wounds were <60% closed. In keeping with this observation, Arg1 activation substantially increased Arg1 levels (*P* < 0.001; **Figures 5C, E**) and reduced iNOS expression (*P* < 0.001; **Figures 5D, F**) in the vaginal epithelium. Finally, Daidzein treatment delivered significant functional improvement to the healed vagina, where the force required to break the daidzein-treated wounds in age rats was comparable to that of young animals (*P* < 0.01; **Figure 5G**). These data thus highlight the potential therapeutic benefit of Arg1 activation in age-related vaginal repair.

## Discussion

Kamfer et al., first demonstrated the induction of the arginase enzymatic system upon skin injury in mice (31). In this context, reparative effects of Arg1 have mostly been linked to its expression by anti-inflammatory macrophages, which dampen inflammation and promote ECM deposition in murine skin wounds (24, 32). Here, macrophage “switching” from pro-inflammatory (“M1”) to anti-inflammatory (“M2”) states is vital for effective tissue repair, with perturbations in this process associated with delayed healing (33–35). While Arg1-expressing macrophages are present in murine wounds, it is important to note that human macrophages do not readily express Arg1 (36). Nevertheless, other cell types in acute (37) and chronic (38) human cutaneous wounds do express Arg1, and Arg1 is required for effective healing in human *ex vivo* skin (25). Indeed, new data suggest that epidermal Arg1 also contributes to effective skin repair in rodents and humans (25). Our data now reveal a direct link between Arg1 and age-associated delayed healing in the vagina, with significant clinical importance given the association between ageing, vaginal prolapse and recurrence following reconstructive surgery (13, 18).

We report that aged rat vaginal wounds show delayed healing and significantly reduced Arg1 expression versus young rat wounds. These data are in line with previous studies showing reduced Arg1 in the dermis and epidermis of aged murine wounds (24, 25), and reduced Arg1 expression in acute wounds of the elderly (23). It is widely recognised that ageing is associated with a decrease in serum 17β-estradiol (39), while estrogen deficiency correlates more convincingly with delayed healing than chronological age (23). Moreover, 17β-estradiol deficiency in young ovariectomised mice delays tissue repair (40). As ovariectomy is similarly accompanied by reduced Arg1 in murine wounds (24) and rabbit vaginal tissue (41), the concomitant age-related decrease in Arg1 may be linked to 17β-estradiol deficiency. This is interesting given the importance of 17β-estradiol in preventing age-related changes to vaginal tissue physiology (42, 43).

While the underlying pathophysiology of POP is not fully understood, it is known that ageing causes weakening of the connective tissues that provide pelvic organ support (44, 45). These connective tissues consist of a dense matrix of ECM proteins, including collagens, elastin, proteoglycans and glycoproteins. Multiple studies have certainly demonstrated alterations in collagen content and organisation, along with a reduction in elastin fibre width and number, in patients with pelvic floor disorders (46–49). Animal models confirm the importance of proper connective tissue function, as deficiency in the elastin fibre protein, fibulin 3, leads to prolapse in aged mice (50).

In our presented work, we used biomechanical testing to assess the tensile strength of the vaginal ECM between young and aged rats. Although no difference in breaking stress was observed in non-injured tissues, significantly less force was required to break the aged wound tissue, demonstrating that the reformed vagina was weaker in the older rats. As Arg1 activation restored breaking stress of aged rat wounds to levels observed in young rat wounds, our data additionally suggest Arg1 may be fundamentally required for ECM reformation following vaginal injury. This is concurrent with previous work showing that macrophage-specific ablation of Arg1 reduces ECM deposition in skin wounds (24). It is also well established that Arg1 catalyses the hydrolysis of L-arginine to urea and L-ornithine, the latter being an important precursor for proline synthesis (51). As proline is required for collagen synthesis (52), it is unsurprising that arginase is so crucially linked to ECM deposition in tissue repair.

Another key factor to consider in ECM deposition and tissue tensile strength during repair is the presence of ECM-degrading enzymes, or MMPs. Excessive MMP production is widely associated with impaired healing in chronic wounds (53–55). Interestingly, our data show that blocking Arg1 in human vaginal epithelial cells leads to upregulation of *MMP2*, *MMP10* and *MMP12*, which are known to degrade collagens, glycoproteins and elastin (56). Moreover, increased Mmp2 is observed in murine skin wounds with Arg1 deficiency (24), again suggesting that Arg1 is required to enable effective ECM deposition during healing.

Alongside being a precursor for proline, L-ornithine is also an important precursor for polyamines, which are involved in a number of wound-relevant processes, including regulating cellular proliferation (48). Indeed, detrimental alterations in polyamine homeostasis are associated with brain trauma and acute kidney injury (57, 58), while high levels of the polyamine, spermine, drive epidermal repair in human *ex vivo* skin wounds (59). Our collective findings demonstrate that blocking Arg1 inhibits migration in human vaginal epithelial cells and reduces epidermal proliferation *in vitro* and *in vivo*, which may in part be due to inhibition of polyamine synthesis.

It is clear that impaired Arg1 is a contributing factor to delayed healing, as Arg1 is downregulated in delayed healing aged wounds (23, 24), and both genetic knockdown and chemical inhibition of Arg1 increase inflammation and delay repair (24, 25). In addition, previous authors have demonstrated L-arginine supplementation improves healing in diabetic skin wounds *in vivo* and may be beneficial in patients with diabetic foot ulcers (60–62). We thus assessed the reparative efficacy of the Arg1 activator, daidzein, in our aged rat vaginal injury model, showing that daidzein treatment significantly accelerated wound closure, increased Arg1 expression and dampened iNOS.

## Conclusion

In summary, our novel data show that Arg1 is an important mediator of vaginal repair, where age-related Arg1 deficiency causes defective healing. We crucially reveal that Arg1 restoration significantly accelerated vaginal healing in an aged model *in vivo*. Essential follow-on studies are now needed to explore the therapeutic validity of targeting Arg1 deficiency to prevent POP recurrence following surgical intervention.

## Data availability statement

The original contributions presented in the study are included in the article/**Supplementary Material**. Further inquiries can be directed to the corresponding author.

## Ethics statement

The animal study was reviewed and approved by The Hebrew University Animal Care and Use Committee.

## Author contributions

HW, MH and OM-Z conceived and designed this study. HW, BR, DS and OM-Z collected and analysed the data. HW drafted the manuscript. MH and OM-Z provided critical review of the manuscript and supervised the study. All authors contributed to the article and reviewed and approved the final manuscript for submission.

## Funding

This work was supported by the Daniel Turnberg Travel Fellowships of the Academy of Medical Sciences and the kind donation of Judy and Sidney Swartz and Dan and Morrine Marantz.

## Acknowledgments

We would like to thank the Daniel Turnberg Travel Fellowships of the Academy of Medical Sciences and the kind donation of Judy and Sidney Swartz and Dan and Morrine Marantz. Dr Wilkinson and Prof Hardman would also like to thank the Daisy Appeal for providing laboratory facilities.

## Conflict of interest

The authors declare that the research was conducted in the absence of any commercial or financial relationships that could be construed as a potential conflict of interest.

## Publisher’s note

All claims expressed in this article are solely those of the authors and do not necessarily represent those of their affiliated organizations, or those of the publisher, the editors and the reviewers. Any product that may be evaluated in this article, or claim that may be made by its manufacturer, is not guaranteed or endorsed by the publisher.
