# Supplementary material for: Epithelial arginase-1 is a key mediator of age-associated delayed healing in vaginal injury
Source: Front Endocrinol (Lausanne). 2022 Aug 11;13:927224. doi: 10.3389/fendo.2022.927224 (PMC9410732; doi:10.3389/fendo.2022.927224)
Supplement: Supplementary file 1 [file Table_1.docx]

| **Gene Name** | **Forward** | **Reverse** |
| --- | --- | --- |
| GAPDH | TGCACCAACTGCTTAGC | GGCATGGACTGTGGTCATGAG |
| YWHAZ | ACTTTTGGTACATTGTGGCTTCAA | CCGCCAGGACAAACCAGTAT |
| ARG1 | TGGACAGACTAGGAATTGGCA | CCAGTCCGTCAACATCAAAACT |
| NOS2 | ATTCACTCAGCTGTGCATCG | TTTAGCTCCAGTTCCCGAAA |
| IL1B | CTCGCCAGTGAAATGATGGCT | GTCGGAGATTCGTAGCTGGAT |
| TNFA | CAGAGGGCCTGTACCTCATC | GGAAGACCCCTCCCAGATAG |
| MMP2 | ATGACAGCTGCACCACTGAG | ATTTGTTGCCCAGGAAAGTG |
| MMP3 | TCAGAACCTTTCCTGGCATC | TTTCCAGGTCCATCAAAACG |
| MMP9 | TTGACAGCGACAAGAAGTGG | GCCATTCACGTCGTCCTTAT |
| MMP10 | TGGACAGAAGATGCATCAGG | TCCCGAAGGAACAGATTTTG |
| MMP12 | ATGCAGCACTTCTTGGGTCT | TCACGGTTCATGTCAGGTGT |
| MMP13 | AACATCCAAAAACGCCAGAC | GGAAGTTCTGGCCAAAATGA |

**Supplementary Table S1. Primer sequences used for qRT-PCR.**
